# Effects of feeding varying levels of mycotoxin-containing corn fines on diet choice and growth performance of nursery pigs

**DOI:** 10.1093/tas/txaf015

**Published:** 2025-02-04

**Authors:** Duncan B Paczosa, Tyler B Chevalier, Sunday A Adedokun, Lan Zheng, Merlin D Lindemann

**Affiliations:** Department of Animal and Food Sciences, University of Kentucky, Lexington, KY 40546, USA; Department of Animal and Food Sciences, University of Kentucky, Lexington, KY 40546, USA; Department of Animal and Food Sciences, University of Kentucky, Lexington, KY 40546, USA; dsm-firmenich, Plainsboro, NJ 08536, USA; Department of Animal and Food Sciences, University of Kentucky, Lexington, KY 40546, USA

**Keywords:** mycotoxin, performance, pigs, preference

## Abstract

Mycotoxins in feed are known to negatively affect growth and other physiological processes in pigs. Two experiments were conducted to investigate the effects of feeding diets with varying levels of mycotoxins and boron (a nutrient reported to mitigate some aspects of mycotoxicosis). Screenings from the 2020 crop year corn contained mycotoxin levels of 23,038 ppb total fumonisins (FUM), 1,446 ppb zearalenone (ZEA), and 5,032 ppb total deoxynivalenol (DON). The corn fines were added to a corn-soybean meal diet formulated to meet or exceed NRC (National Research Council. 2012. Nutrient requirements of swine. 11th Revised Edition. Washington, DC: The National Academies Press. doi:10.17226/13298) nutrient requirements as a replacement for corn at 0, 10, and 20% for Diets 1 to 3. Diets 4 to 6 were Diets 1 to 3, respectively, plus 40 ppm boron from sodium tetraborate decahydrate. Diets 3 and 6 were formulated to approximate the guidance level of fumonisin and the advisory level of DON stated by the FDA. Exp. 1 used 48 crossbred pigs (initial body weight [BW] = 9.18 kg ± 0.12 kg) blocked by sex and BW and randomly allotted to 1 of 3 treatment comparisons: Comparison 1) Diet 1 vs. Diet 2; Comparison 2) Diet 1 vs. Diet 3; and Comparison 3) Diet 2 vs. Diet 3. There were 4 replicates (4 pigs/pen) for the 21-d preference trial. Exp. 2 used 144 crossbred pigs (mean initial BW = 10.20 ± 0.23 kg) blocked by sex and BW and randomly allotted to diets for a total of 6 replicates (4 pigs/pen) for a 21-d growth trial. On d 21, serum was collected from the heaviest and lightest pig in each pen for clinical chemistry assessment. Exp. 1 demonstrated the barrows’ ability to discern between diets in Comparisons 2 and 3 (*P* < 0.01) for each week while gilts only started to exhibit that ability during Week 3 for Comparison 2 (*P* = 0.06). Increasing mycotoxin levels in Exp. 2 had no effect on overall ADG, ADFI, and G:F (*P* = 0.16, 0.53, and 0.92, respectively). The increasing mycotoxin levels affected serum glucose and cholesterol (*P* = 0.03, and *P *< 0.01, respectively). There was no effect of boron on the same performance measures (*P* = 0.81, 0.59, and 0.76, respectively) although it did lower serum glucose (*P* = 0.02). In conclusion, pigs can differentiate and choose between diets containing these mixed mycotoxins but when not given a choice, the pigs do not necessarily have different growth performance using the particular mycotoxins and concentrations within the framework of this assessment.

## Teaser

1. Pigs can discern between diets containing different levels of mycotoxins and favor the diet with lower concentrations of mycotoxins.

2. Increasing fumonisins and deoxynivalenol concentrations to the FDA guidance and advisory levels did not decrease pig performance.

## INTRODUCTION

Mycotoxins are secondary metabolites of filamentous fungi (molds) that, when ingested by animals, can cause a variety of adverse physiological responses. Mycotoxin contaminated feed may result in feed refusal, digestive problems, nervous system problems, reproductive problems, immune suppression, organ damage, and cancer (NRC, 2012). These responses on animal health and reproduction have been estimated to result in an economic loss of $1.4 billion annually in the United States (CAST, 2003) from three mycotoxins (aflatoxins, deoxynivalenol, and fumonisins). In the dsm-firmenich US corn mycotoxin report (dsm, 2024) that analyzed over 250 corn samples of the 2023 harvested crop from 20 states for 6 major mycotoxin groups, 94% of the samples were above the detection limit with 78% reported to contain more than 1 mycotoxin.

It is known that mycotoxins fed to pigs results in unsatisfactory performance and illnesses. Understanding the relationship between the mycotoxin interactions as well as the time-dose relationship may result in opportunities to improve pig health and production. Knowing the contaminants and contamination levels of the feed results in several intervention opportunities. One opportunity is screening the corn for fines which often contain the highest concentrations of mycotoxins. Mycotoxin binders can also be added to the diet to prevent absorption of the mycotoxin as well as enzymes to detoxify the mycotoxin. A recent study by Taranu et al. (2011) reported that the addition of boron as calcium fructoborate ameliorated the reduction in performance and reduced the exacerbated cellular immune response caused by mycotoxins.

The hypothesis of the current study was that including mycotoxin-containing corn fines would adversely affect nursery pig performance. The specific objective was to investigate three levels of mycotoxin-contaminated diets and to evaluate boron as a possible mitigant to the anticipated adverse performance.

## MATERIALS AND METHODS

This experiment was carried out in an environmentally controlled room at the University of Kentucky Swine Research Center. The experiment was conducted under protocols approved by the Institutional Animal Care and Use Committee of the University of Kentucky and within the husbandry guidelines for the care and use of agricultural animals in research and teaching (Ag Guide, 2020).

### Diets

The 2020 corn harvest at the University of Kentucky Research Farm was screened to isolate corn fines from the corn using the Kwik-Kleen grain cleaner (Model No. 772, Norwood Sales Inc., Horace, ND) with a screen slot of 6/64” by 3/4”. The fines from a pre-study screening were analyzed for concentrations of mycotoxins and nutrients (Tables 1 and 2). The corn fines used in this study contained mycotoxin levels that were analyzed to be 23,038 ppb total fumonisin (FUM), 1,446 ppb zearalenone (ZEA), and 5,032 ppb total deoxynivalenol (DON). Experimental diets (Table 3) were formulated to meet or exceed the NRC (2012) requirement estimates relative to body weight (BW). To evaluate the effects of feeding corn fines (screenings) containing mycotoxin levels at the FDA guidance (fumonisins) and advisory (deoxynivalenol) levels (FDA, 2001, 2010), dietary corn fines were added to a corn-soybean meal-based basal diet at concentrations of 0, 10, and 20% of the diet (Diets 1 to 3 respectively; Table 4) for a target level of about 5,000 ppb FUM, 500 ppb ZEA, and 1,000 ppb total DON for Diet 3. To minimize potential differences in non-treatment components of the diet, two basal diets were mixed according to Table 3. The first basal diet served as the control diet (0% fines) whereas the second basal diet served as Diet 3 (20% fines). Diet 3 was made by replacing 20% of the corn with corn fines. By blending appropriate amounts of Diets 1 and 3 (i.e., 50:50 blend), an intermediate diet (Diet 2, 10% fines) was created.

**Table 1.** Effects of screening corn on mycotoxin content^1^^,^^2^

Mycotoxin, ppb	DL	Uncleaned	Cleaned	Fines
Fumonisin B_1_	200	2,055	597	17,471
Fumonisin B_2_	200	537	<200	4,447
Zearalenone	100	134	112	662
Deoxynivalenol	200	1,366	961	5,971

^1^Corn was analyzed by the Veterinary Diagnostic Laboratory at North Dakota State University (Fargo ND).

^2^Additional mycotoxins assayed which were below the detection level (DL) were: aflatoxin B_1_ (DL-20 ppb), aflatoxin B_2_ (DL-20 ppb), aflatoxin G_1_ (DL-20 ppb), aflatoxin G_2_ (DL-20 ppb), HT-2 toxin (DL-200 ppb), T2 toxin (DL-20 ppb), diacetoxyscirpenol (DL-200 ppb), ochratoxin A (DL-20 ppb), sterigmatocystin (DL-20 ppb), 15-acetyldeoxynivalenol (DL-200 ppb), 3-acetyldeoxynivalenol (DL-200 ppb).

**Table 2.** Effects of screening corn on nutrient content^1^^,^^2^

	Uncleaned	Cleaned	Fines	SEM
CP, %	8.23^a^^,^^b^	8.09^b^	9.02^a^	0.149
GE, kcal/kg	3,877	3,885	3,856	5.706
Crude fat, %	2.90^a^	2.74^a^	1.64^b^	0.133
NDF, %	8.18^b^	7.96^b^	14.82^a^	0.327
ADF, %	2.82^b^	2.66^b^	4.42^a^	0.045
Calcium, %	0.009	0.005	0.013	0.002
Phosphorus, %	0.30	0.31	0.31	0.008
Amino acids, %				
Taurine	0.095^b^	0.085^b^	0.120^a^	0.003
Hydroxyproline	0.040	0.035	0.065	0.013
Aspartic Acid	0.570^a^^,^^b^	0.540^b^	0.615^a^	0.009
Threonine	0.275^b^	0.260^b^	0.315^a^	0.005
Serine	0.355^b^	0.350^b^	0.375^a^	0.003
Glutamic Acid	1.435	1.425	1.445	0.010
Proline	0.685	0.690	0.685	0.008
Glycine	0.310	0.285	0.340	0.016
Alanine	0.585	0.575	0.580	0.009
Cysteine	0.155	0.145	0.160	0.003
Valine	0.375^a^^,^^b^	0.360^b^	0.410^a^	0.008
Methionine	0.145	0.130	0.140	0.003
Isoleucine	0.290	0.290	0.310	< 0.001
Leucine	0.940	0.945	0.940	0.005
Tyrosine	0.220^a^^,^^b^	0.185^b^	0.240^a^	0.009
Phenylalanine	0.395	0.390	0.405	0.003
Hydroxylysine	0.020	0.020	0.035	0.003
Ornithine	0.010	0.010	0.010	0.000
Lysine	0.260	0.240	0.285	0.009
Histidine	0.215	0.210	0.210	0.003
Arginine	0.340	0.310	0.340	0.006
Tryptophan	0.050	0.050	0.050	< 0.001

^1^CP = crude protein, GE = gross energy, NDF = neutral detergent fiber, ADF = acid detergent fiber.

^2^All assays conducted in duplicates.

^a^,

^b^Means within a row without a common superscript differ (*P *< 0.05).

**Table 3.** Composition of the basal diet (%, as-fed basis)

Ingredient, %	Diet 1	Diet 3
Corn	59.55	39.55
Corn fines	0.00	20.00
Soybean meal	35.00	35.00
Choice white grease	2.00	2.00
L-Lysine	0.28	0.28
DL-Methionine	0.19	0.19
L-Threonine	0.11	0.11
Dicalcium phosphate	1.20	1.20
Limestone	0.90	0.90
Salt	0.50	0.50
Copper sulfate	0.08	0.08
Trace-mineral premix^1^	0.10	0.10
Vitamin premix^2^	0.08	0.08
Santoquin^3^	0.02	0.02
Total:	100.00	100.00
Calculated nutrient composition^4^		
Metabolizable energy, kcal/kg	3360	3360
Crude protein, %	22.07	22.07
SID Lysine, %^5^	1.25	1.25
Calcium, %	0.73	0.73
Total Phosphorus, %	0.63	0.63
STTD Phosphorus, %^6^	0.39	0.39
Analyzed nutrient composition^7^		
Gross energy, kcal/kg	4077	4164
Crude protein, %	22.34	21.41
Calcium, %	0.89	0.65
Total phosphorus, %	0.62	0.58

^1^Provided the following per kg of diet: Zn, 131 mg as ZnO; Fe, 131 mg as FeSO_4_·H2O; Mn, 45 mg as MnO; Cu, 13 mg as CuSO_4_·5H_2_O; I, 1.5 mg as CaI_2_O_6_; Co, 0.23 mg as CoCO_3_; Se, 0.28 mg as NaSeO_3_.

^2^Provided the following per kg of diet: vitamin A, 6,600 IU; vitamin D_3_, 880 IU; vitamin E, 44 IU; vitamin K (as menadione sodium bisulfite complex), 6.4 mg; thiamin, 4.0 mg; riboflavin, 8.8 mg; pyridoxine, 4.4 mg; vitamin B_12_, 33 μg; folic acid, 1.3 mg; niacin, 44 mg; pantothenic acid, 22 mg; D–biotin, 0.22 mg.

^3^Provided 130 mg ethoxyquin per kg of diet (Novus International, St. Louis, MO).

^4^Values for metabolizable energy, crude protein, SID lysine, calcium, total phosphorus, and STTD phosphorus were obtained from the feedstuff values listed in the NRC (2012).

^5^SID = standardized ileal digestible basis.

^6^STTD = standardized total tract digestible basis.

^7^All assays conducted in duplicates.

**Table 4.** Mycotoxin content of the diets^1^^,^^2^

	Detection Level	Cleaned Corn	Corn Fines	Diet 1 (0%)	Diet 2 (10%)	Diet 3 (20%)
Mycotoxin	ppb	ppb	ppb	ppb	ppb	ppb
Fumonisin B_1_	200	953	16,597	642	1,787	3,062
Fumonisin B_2_	200	297	4,999	< 200	592	839
Fumonisin B_3_	200	< 200	1,442	< 200	203	286
Zearalenone	100	< 100	1,446	< 100	306	304
Deoxynivalenol	200	835	4,743	479	898	1,245
15-ADON	200	< 200	289	< 200	< 200	< 200

^1^Diets 1 to 3 were Diets 4 to 6, respectively, plus 40 ppm boron.

^2^Additional mycotoxins assayed which were below the detection level (DL) were: aflatoxin B_1_ (DL-20 ppb), aflatoxin B_2_ (DL-20 ppb), aflatoxin G_1_ (DL-20 ppb), aflatoxin G_2_ (DL-20 ppb), HT-2 toxin (DL-200 ppb), T2 toxin (DL-20 ppb), diacetoxyscirpenol (DL-200 ppb), Ochratoxin A (DL-20 ppb), Sterigmatocystin (DL-20 ppb), 3-acetyldeoxynivalenol (DL-200 ppb).

Three additional treatments (Diets 4, 5, and 6) were used to evaluate a potential dietary mycotoxin mitigant. Diets 4, 5, and 6 were each made by taking a portion of Diets 1 to 3, respectively, and adding an appropriate amount of sodium tetraborate decahydrate (11.34% B) to result in 40 mg B/kg diet.

### Animals, Experimental Design, and Housing

#### Experiment 1.

A total of 48 crossbred pigs (Yorkshire × Landrace × Large White; 24 barrows and 24 gilts; mean initial BW: 9.18 ± 0.12 kg; 1 wk post weaning) were blocked by sex and BW, and then randomly allotted within block to one of 3 treatment comparisons for a preference trial using the Experimental Animal Allotment Program (Kim and Lindemann, 2007). Pigs were housed in totally-slatted nursery pens (1.22 × 2.44 m^2^) containing 4 pigs/pen with 4 replicate pens. Each pen contained two nipple waterers and two feeders with each feeder containing one of three diets. The 3 treatment comparisons were: Comparison 1) Diet 1 vs. Diet 2; Comparison 2) Diet 1 vs. Diet 3; and Comparison 3) Diet 2 vs. Diet 3. To avoid the possibility that pigs became accustomed to eating in a particular feeder location, dietary feeder locations were switched every Monday, Wednesday, and Friday throughout the duration of the experiment. In total, the experiment lasted for 3 wk.

#### Experiment 2.

A total of 144 crossbred pigs (Yorkshire × Landrace × Large White; 72 barrows and 72 gilts; initial BW: 10.20 ± 0.23 kg; 1 wk post weaning) were blocked by sex and BW, and then randomly allotted within block to one of six dietary treatments for a performance trial using the Experimental Animal Allotment Program (Kim and Lindemann, 2007). The six dietary treatments were a result of a 3 (corn fines inclusion level) × 2 (boron addition) factorial arrangement. Pigs were housed in totally-slatted nursery pens (1.22 × 2.44 m^2^) containing 4 pigs/pen with 6 replicate pens. Each pen contained two nipple waterers and one feeder. In total, the experiment lasted for 3 wk. To determine serum clinical chemistry profiles, a total of 2 pigs/pen were selected for blood sampling on d 21.

Throughout the duration of both experiments, animal care providers were observing for possible vomiting (caused by DON) and swollen vulvas in gilts (caused by ZEA).

### Data Collection and Laboratory Analysis

Individual pig BW and feed disappearance were recorded every week to calculate average daily gain (ADG), average daily feed intake (ADFI), gain to feed ratio (G:F). A total of 2 pigs per pen in experiment 2 were selected at the end of Wk 3 for blood collection. Pigs used for blood collection were determined by selecting the smallest and heaviest pigs within a pen.

Blood samples were collected via vena cava puncture using a needle holder and vacutainer tube with polymer gel (Becton, Dickinson, and Company, Franklin Lakes, NJ) for serum separation. Following collection of blood, samples were allowed to clot at room temperature for 30 to 45 min. Serum was obtained by centrifugation of the blood samples at 2500 × g and 4 °C for 20 min. Serum samples were stored at −80 °C until the chemical assays were performed.

Aliquots of serum were sent to the University of Kentucky Veterinary Diagnostic Laboratory (Lexington, KY) where a porcine serum chemistry panel was performed using an Alfa Wassermann Vet Axel chemistry analyzer (Alfa Wassermann Diagnostic Technologies LLC., West Caldwell, NJ).

Diet samples were analyzed for nitrogen (method 990.03; AOAC Int., 2006), calcium and phosphorus (method 993.14; AOAC Int., 2006) at the Experiment Station Chemical Laboratories at the University of Missouri (Columbia, MO). Gross Energy was analyzed using a bomb calorimeter (Parr adiabatic bomb calorimeter, model 6200, Parr Instruments, Moline, IL) according to the manufacturers instructions with the calibration standard being benzoic acid.

Samples of individual diets, corn, and corn fines were obtained at the feed mill following diet mixing. Individual diets, fines, and corn samples were sent to North Dakota State University Veterinary Diagnostic Laboratory (Fargo, ND) for mycotoxin analysis by liquid chromatography- tandem mass spectrometry (LC/MS/MS). The mycotoxin panel and the detection levels (DL) were: aflatoxin B_1_ (DL-20 ppb), aflatoxin B_2_ (DL-20 ppb), aflatoxin G1 (DL-20 ppb), aflatoxin G2 (DL-20 ppb), fumonisin B_1_ (DL-200 ppb), fumonisin B_2_ (DL-200 ppb), fumonisin B_3_ (DL-200 ppb), HT-2 toxin (DL-200 ppb), T2 toxin (DL-20 ppb), DAS (DL-200 ppb), ochratoxin A (DL-20 ppb), sterigmatocystin (DL-20 ppb), zearalenone (DL-100 ppb), deoxynivalenol (DL-200 ppb), 15-ADON (DL-200 ppb) and 3-ADON (DL-200 ppb).

### Statistical Analysis

Preference data from Exp.1 was evaluated via T-test utilizing Microsoft Excel (Redmond, WA), with the pen as the experimental unit with a level of significance set at *P* < 0.05.

Experiment 2 was designed and analyzed as a randomized complete block design for a factorial arrangement of corn fines inclusion level and boron addition. An analysis of variance was conducted by using the General Linear Model (Proc GLM) procedure of the Statistical Analysis System (SAS, version 9.4, Cary, NC). The statistical model for the growth performance included the terms for the main effects of fines and boron, as well as the fines by boron interaction, sex, and replicate (sex). The statistical model for the clinical chemistry data also included a term for the weight of the pig (i.e., heavy or light pig). Orthogonal polynomial contrasts were used to determine linear and quadratic effects with increasing dietary levels of corn fines. Mean separations were accomplished by the PDIFF procedure for performance data when *P* < 0.05 and for clinical chemistry data when *P* < 0.10 for the interaction of fines by boron. Performance data analysis utilized the pen as the experimental unit and clinical chemistry data analysis utilized the individual pig as the experimental unit with the level of significance set at *P* < 0.05.

## RESULTS AND DISCUSSION

### Corn Screening – A Pre-Study Evaluation of the Process

Corn fines from an initial screening prior to the experiments were analyzed for mycotoxin and nutrient content. The efficacy of removing fines is shown in Table 1 resulting in the clean corn to contain fumonisin B_1_ (FB_1_) concentrations of 597 ppb (originally 2,055 ppb) removing approximately 71% of the detected mycotoxin. This removal is higher than reported by Yoder et al. (2021) and Scudamore and Patel (2000), who found a 42% and 32% reduction in fumonisin, respectively. The cleaned corn concentration of fumonisin B_2_ (FB_2_), ZEA, and DON, respectively, were < 200, 112, and 961 ppb and before screening were 537, 134, and 1,366 ppb. The corn fines were highly concentrated with FB_1_, FB_2_, ZEA, and DON reported at 17,471, 4,447, 662, and 5,971 ppb, respectively.

The nutrient composition of the uncleaned and cleaned corn, as well as the fines are presented in Table 2. All measurements were compared to available values for corn, yellow dent, in the NRC (2012). Corn fines reported to be at least one SD over the reported NRC (2012) mean for corn itself are acid detergent fiber and neutral detergent fiber which is different (*P* < 0.05) from both uncleaned and cleaned corn. This increase in fiber may result in a decrease of metabolizable energy for the pig compared to the cleaned corn. This data suggests that screening corn is a viable option to remove mycotoxins without materially affecting the nutrient value. The process will lower the concentration of mycotoxins in the corn to make it safer for consumption.

### Diet Analysis

The specific corn fines used in Exp. 1 and 2 were obtained by a separate screening of the 2020 crop year and 2.34% (14,700 lbs of fines from 628,992 lbs of corn) was removed as corn fines. The corn fines contained mycotoxin levels of 16,597 ppb FB_1_, 4,999 ppb FB_2_, 1,442 ppb FB_3_, 1,446 ppb ZEA, 4,743 ppb DON, and 289 ppb 15-ADON (Table 4). As previously stated, fines replaced 20% of the corn to mix Diet 3 (20% corn fines) to obtain levels of about 5,000 ppb total FUM, 500 ppb total ZEA, and 1,000 ppb total DON which are mycotoxin levels at the FDA guidance (fumonisins) and advisory (deoxynivalenol) levels (FDA, 2001, 2010). The mean content of Diet 3 was 4,187 ppb total FUM, 304 ppb ZEA, and 1,245 ppb DON. Diet 2 was blended at proportion with half Diet 1 and half Diet 3 to be an intermediary diet. This resulted in total FUM and DON to roughly be the mean concentration of Diets 1 and 3. Zearalenone in Diet 2 is reported to be higher than Diet 3, this must be the result of lab or sampling variation because it is not physically possible due to the method of mixing.

#### Experiment 1.

Dual choice tests were conducted to evaluate the ability of barrows and gilts to discern between diets in three dietary comparisons. Many preference tests have been done to establish whether pigs show a preference when given a choice. Palatability can be affected by the nature of the feedstuff, inclusion rate, and freshness (Solà-Oriol et al., 2011). Critical times to ensure a feedstuff has a high palatability are at times of stress like weaning or moving barn location. Weaning may be the most crucial because pigs are also adjusting to a new source of feed. During the duration of this trial, no pigs were removed and performance was good being 657, 691, and 640 g/d for Comparisons 1 to 3, respectively, which exceeds the daily gain listed in NRC (2012) for pigs weighing 11 to 25 kg.

##### Comparison 1.

Table 5 shows the results of the three comparisons made during this experiment. During Comparison 1 with either 0% (Diet 1) or 10% (Diet 2) fines, there was no statistical difference observed (P > 0.05), however the pigs showed an inclination choosing the diet containing 0% fines about 17% more than the diet with 10% fines within the first week that stayed consistent throughout the duration of the experiment (cumulatively, 59.3% vs 40.7%). This inclination, when viewed with the next two comparisons, revealed a pattern.

**Table 5.** Feed preference of pigs (Exp. 1) fed diets with different levels of mycotoxin containing corn fines

Comparison 1					
	Feed intake, kg/d			Diet 1 vs. Diet 2 0% vs. 10% Fines	
Days	0%	10%	SEM	For the period, % consumed	Cumulative consumption
0 to 7	0.407	0.297	0.071	58.5 vs. 41.5	
7 to 14	0.572	0.386	0.103	58.9 vs. 41.1	58.8 vs. 41.2
14 to 21	0.735	0.431	0.113	58.3 vs. 41.7	59.3 vs. 40.7
				Barrows:	58.94 vs. 41.06
				Gilts:	59.74 vs. 40.26
Comparison 2					
	Feed intake, kg/d			Diet 1 vs. Diet 3 0% vs. 20% Fines	
Days	0%	20%	SEM	For the period, % consumed	Cumulative consumption
0 to 7	0.472	0.219	0.066	69.1 vs. 30.9*	
7 to 14	0.788	0.197	0.156	80.4 vs. 19.6**	75.9 vs. 24.1**
14 to 21	0.979	0.342	0.21	73.5 vs. 26.5**	74.7 vs. 25.3**
				Barrows:	84.66 vs 15.34**
				Gilts:	64.75 vs. 35.25*
Comparison 3					
	Feed intake, kg/d			Diet 2 vs. Diet 3 10 vs. 20% Fines	
Days	10%	20%	SEM	For the period, % consumed	Cumulative consumption
0 to 7	0.494	0.161	0.209	76.5 vs. 23.5**	
7 to 14	0.657	0.272	0.229	71.2 vs. 28.8**	73.3 vs. 26.7**
14 to 21	0.822	0.347	0.216	70.8 vs. 29.2*	72.2 vs. 27.8**
				Barrows:	78.14 vs. 21.86**
				Gilts:	66.35 vs. 33.65

^1^Each mean represents 4 observations (2 barrow and 2 gilt replicates) per treatment comparison (*P < 0.05; **P < 0.01).

##### Comparison 2.

The second comparison consisted of diets with either 0% (Diet 1) or 20% (Diet 3) corn fines. Pigs were able to significantly discern between diets within the first week (*P* < 0.05), choosing Diet 1 38% more compared to Diet 3. Weeks 2 and 3 resulted in the same preference of pigs choosing Diet 1 over Diet 3 (cumulatively, 74.7% vs 25.3%, *P* < 0.01). Barrows consumed the diet with no fines 84.66% of the time while gilts were below that at 64.75% (*P* < 0.01 and 0.05, respectively). The difference in the ability to discern between diets may be a result of the DON in the diet to which males are more sensitive (Kamle et al., 2022).

##### Comparison 3.

The third comparison was of 10% (Diet 2) and 20% (Diet 3) corn fines. As with Comparison 1 and 2, pigs preferred the diet with the lowest amount of mycotoxin-containing corn fines (i.e., Diet 2) during each week and cumulatively. Overall, consumption of Diet 2 vs Diet 3 was 72.2% vs 27.8%, respectively (*P* < 0.01). This comparison also showed a greater discerning ability for Diet 2 vs Diet 3 by the barrows (78.1% vs 21.9%, *P* < 0.01) compared to the gilts (66.4% vs 33.6%, *P* > 0.05).

This data shows pigs can discern between diets consisting of different concentrations of mycotoxin-containing corn fines. These results are consistent with preference tests utilizing corn contaminated with the same 3 mycotoxins that has been done previously in our lab (Jang et al., 2018) wherein the preference for uncontaminated diets was as much as 95.3% vs 4.7% over contaminated diets. Taste allows pigs the ability to sense nutrients and harmful substances, guiding the selection of nutritious feeds and avoiding toxins (Klasing and Humphrey, 2009). If given a choice, pigs will choose the diet with lower amounts of mycotoxins.

### Experiment 2

#### Performance.

Throughout the duration of the performance experiment, no pigs were removed. Table 6 presents pig BW, ADG, ADFI, and G:F ratio. Despite the fact that there were sporadic instances where a performance measure differed during an individual week, there were no patterns of response and no overall effects of fines or inorganic boron (as sodium tetraborate decahydrate) supplementation on these performance parameters. Taranu et al. (2011) found the addition of calcium fructoborate to diets, fed to 16 pigs, contaminated with 1,790 ppb DON, 20 ppb ZEA, 0 to 6 ppb fumonisins, and 90 ppb T-2/H-2 toxin ameliorated most of the decrease in performance and the exacerbated cellular immune responses induced by the mycotoxins.

**Table 6.** Growth performance of pigs (Exp. 2) fed increasing levels of mycotoxin containing corn fines^1^

Boron, ppm	0			40				*P-*value		
Fines, %	0	10	20	0	10	20	SEM	Boron	Fines	Boron*Fines
Body weight, kg										
d 0	9.98	10.00	9.98	10.03	10.04	10.03	0.037	0.17	0.89	0.95
d 7	13.80^ab^	13.50^bc^	13.51^bc^	13.79^ab^	13.96^a^	13.35^c^	0.157	0.34	0.03 ^L^	0.04
d 14	19.28^a^	18.50^c^	18.89^abc^	19.22^ab^	19.28^a^	18.60^bc^	0.243	0.35	0.04 ^L^	0.02
d 21	24.98	24.19	24.42	24.71	24.80	24.38	0.367	0.68	0.27	0.24
Average daily gain, kg										
d 0 to 7	0.55^ab^	0.50^dc^	0.50^bcd^	0.54^abc^	0.56^a^	0.47^d^	0.021	0.58	0.02 ^L^	0.02
d 7 to 14	0.78	0.72	0.77	0.78	0.76	0.75	0.026	0.66	0.09	0.25
d 14 to 21	0.81	0.81	0.79	0.78	0.79	0.83	0.023	0.73	0.83	0.24
d 0 to 21	0.71	0.67	0.69	0.70	0.70	0.68	0.017	0.77	0.23	0.19
Average daily feed intake, kg										
d 0 to 7	0.79^ab^	0.72^b^	0.74^b^	0.75^ab^	0.80^a^	0.79^ab^	0.030	0.09	0.83	0.03
d 7 to 14	1.10	1.05	1.08	1.08	1.12	1.03	0.040	0.99	0.53	0.18
d 14 to 21	1.28	1.15	1.28	1.24	1.27	1.27	0.045	0.43	0.20	0.06
d 0 to 21	1.06^a^	0.97^b^	1.03^ab^	1.02^ab^	1.06^a^	1.03^ab^	0.031	0.35	0.63	0.04
Gain:Feed ratio										
d 0 to 7	0.69^a^	0.69^a^	0.69^a^	0.71^a^	0.70^a^	0.60^b^	0.023	0.28	0.02 ^L^	0.03
d 7 to 14	0.71	0.68	0.72	0.72	0.68	0.73	0.022	0.63	0.03 ^L^	0.88
d 14 to 21	0.64^b^	0.70^a^	0.62^b^	0.63^b^	0.62^b^	0.65^ab^	0.027	0.27	0.31	0.04
d 0 to 21	0.68	0.69	0.68	0.69	0.67	0.66	0.018	0.39	0.58	0.43

^1^Each value represents the mean of 6 replicates (4 pigs/pen).

^a,b,c^Means within a row without a common superscript differ (*P *< 0.05).

^L^Linear effect for dietary inclusion of fines (*P *< 0.05).

A study with similar mycotoxins conducted by Wilson et al. (2022) found that increasing the concentration of deoxynivalenol and zearalenone and decreasing fumonisins from 600, 400, and 250 ppb to 3570, 624, and 120 ppb, respectively, decreased ADG, ADFI, and G:F ratio (P < 0.05). Frobose et al. (2015) observed the same negative effect on ADG, ADFI, and G:F when DON, fumonisin, and ZEA were naturally contaminated in the diet (4600, 2000, and 500 ppb, respectively). The level of fines in this study did not elicit a response in performance measures as was observed by Wilson et al. (2022) and Frobose et al. (2015). While these two studies contain the same mycotoxins as the current study, the concentrations of the mycotoxins differ and studies with co-contamination of mycotoxins can result in quite different physiological responses (Alassane-Kpembi et al., 2017).

#### Clinical chemistry.

Serum was collected for clinical chemistry on the final day of study (d 21). Pig selection consisted of the heavy and light pigs within a pen to measure any possible discrepancies between pig weight and analyte; it was hypothesized that the light weight pigs may have been affected more by the mycotoxins in the diets. Values are reported in Tables 7–9 as the mean treatment analyte measurements and the measurements between the heavy and light pigs within each pen in the different treatments. Analyte values were compared to the Merck Index (2006) reference range with some analytes measuring just outside the reference range.

**Table 7.** Mineral analyte clinical chemistry for pigs (Exp. 2) fed varying levels of mycotoxin containing corn fines^1^^,^^2^

		0 ppm Boron			40 ppm Boron				*P*-values		
Item	Merck Reference range	Diet 1 (0% Fines)	Diet 2 (10% Fines)	Diet 3 (20% Fines)	Diet 4 (0% Fines)	Diet 5 (10% Fines)	Diet 6 (20% Fines)	SEM^3^	Boron	Fines	Fines*Boron
Sodium, mmol/L	139 to 153	142.3	142.3	140.6	143.3	142.1	141.1	0.53	0.33	0.00 ^L^	0.49
Light pig		142.8	143.2	140.3	143.2	142.8	141.2	0.67	0.62	0.00 ^L^	0.69
Heavy pig		141.7	141.5	140.8	143.3	141.3	141.0	0.78	0.39	0.14	0.47
Potassium^4^, mmol/L	4.4 to 6.5	6.20	6.31	5.78	6.27	6.08	6.04	0.17	0.83	0.13	0.35
Light pig		6.35	6.51	5.88	6.40	6.10	6.35	0.28	0.89	0.64	0.32
Heavy pig		6.05	6.10	5.68	6.13	6.05	5.73	0.17	0.84	0.06 ^L^	0.92
Chloride, mmol/L	97 to 106	102.0	102.3	101.7	102.6	102.1	102.5	0.48	0.32	0.91	0.50
Light pig		102.0	102.7	101.7	103.7	102.5	102.2	0.83	0.34	0.53	0.54
Heavy pig		102.0	102.0	101.7	101.5	101.7	102.8	0.53	0.80	0.60	0.24
Calcium, mg/dL	9.3 to 11.5	11.74	11.78	11.68	11.78	11.36	11.28	0.16	0.04	0.21	0.27
Light pig		11.85	11.83	11.83	11.98	11.20	11.47	0.28	0.22	0.36	0.39
Heavy pig		11.63	11.73	11.53	11.57	11.52	11.08	0.15	0.07	0.10	0.47
Phosphorus, mg/dL	5.5 to 9.3	9.97	9.82	9.56	9.67	9.77	9.61	0.20	0.56	0.46	0.68
Light pig		9.75	9.68	9.48	9.43	9.67	9.57	0.35	0.77	0.91	0.84
Heavy pig		10.18	9.95	9.63	9.90	9.87	9.65	0.22	0.53	0.21	0.79
Magnesium, mg/dL	2.3 to 3.5	2.69	2.77	2.58	2.81	2.63	2.58	0.07	0.96	0.05 ^L^	0.20
Light pig		2.77	2.82	2.62	2.73	2.73	2.58	0.11	0.59	0.27	0.97
Heavy pig		2.62^b^	2.72^ab^	2.53^b^	2.88^a^	2.53^b^	2.58^b^	0.08	0.52	0.09 ^L^	0.04

^1^Each main effect represents the mean of 6 replicates (2 pigs/pen) for blood collected on day 21.

^2^In each pen, the heavy and light pig were selected for blood collection at week 3.

^3^SEM represent highest value.

^4^Light and heavy pig differ (*P *< 0.05).

^a,b^Means within a row without a common superscript differ (*P *< 0.05) when the fines × boron interaction is *P <* 0.10.

^L^Linear effect for dietary inclusion of fines (*P < 0.05*).

Table 7 reports the effects that increasing levels of mycotoxin-containing corn fines with two levels of boron have on mineral analytes. The addition of boron showed no effect among the mineral analytes except for Ca (*P* = 0.04). The increasing level of fines resulted in a linear decrease in Na concentration as a whole and for the light pig (*P* < 0.05). Deoxynivalenol has been reported to inhibit the sodium glucose-linked transporter 1 (SGLT1) in chickens (Awad et al., 2007). This inhibitory action agrees with Maresca et al. (2002) who reported that low concentrations of DON to human intestinal cells the SGLT1 appeared to be the most sensitive followed by the d-fructose transporter (GLUT5). The mechanism by which DON suppresses the activity of the SGLT1 is unknown. Yet, there was no significant interaction between the heavy and light pig. Serum Mg concentration showed an effect as fines increased and for the interaction for fines and boron for the heavy pig (*P* < 0.05). Potassium was the only mineral analyte reported to have an interaction between the heavy and light pig (*P* = 0.05), with the light pigs having higher serum concentrations.

Table 8 reports protein and energy analytes in relation to increasing corn fines and two levels of boron. The data observed in this study indicates a linear increase in serum glucose as fines increase (*P* = 0.03). This may not be in agreement with the effect that DON has been reported to have on the SGLT1 transporter. If the SGLT1 transporter is inhibited by DON as indicated by Maresca et al. (2002) and Awad et al. (2007) this would seemingly cause glucose concentrations to decrease. However, it should be noted that while the effects of Maresca et al. (2002) and Awad et al. (2007) may seem to provide different responses, both studies were in vitro studies with a single mycotoxin and with cells from non-porcine species, thus the results are not necessarily in conflict with current observations. The addition of boron significantly increased serum glucose concentration (*P* = 0.02). There is evidence to suggest that boron may affect the levels of glucose by reducing the amount of insulin needed to maintain glucose concentrations (Bakken and Hunt, 2003). Cholesterol was also detected to have a significant linear decrease for overall cholesterol as fines increased in the diet. The increase in dietary fines would have resulted in an increase in total dietary fiber; interestingly, a meta-analysis by Brown et al. (1999) analyzed 67 controlled clinical trials that indicated that high soluble fiber decreases total and low-density lipoprotein cholesterol. There was also a linear decrease for heavy pigs for total protein, albumin, and cholesterol. These results disagree with Gbore (2009) who reported higher serum total protein, albumin, and cholesterol in the low FB_1_ inclusion diet compared to the two higher inclusion levels of FB_1_ (5,000, 10,000, and 15,000 ppb FB_1_, respectively). Dänicke et al. (2004) reported no significant treatment effects on total protein and albumin when DON was at levels from 0 to 2,740 ppb.

**Table 8.** Protein and energy analyte clinical chemistry for pigs (Exp. 2) fed varying levels of mycotoxin containing corn fines^1^^,^^2^

		0 ppm Boron			40 ppm Boron				*P*-values		
Item	Merck Reference range	Diet 1 (0% Fines)	Diet 2 (10% Fines)	Diet 3 (20% Fines)	Diet 4 (0% Fines)	Diet 5 (10% Fines)	Diet 6 (20% Fines)	SEM^3^	Boron	Fines	Fines*Boron
Total protein^4^, g/dL	5.8 to 8.3	5.36	5.33	5.08	5.25	5.31	5.28	0.08	0.75	0.21	0.18
Light pig		5.52^a,b^	5.45^a,b^	5.13^b^	5.13^b^	5.42^a,b^	5.67^a^	0.14	0.75	0.75	0.02
Heavy pig		5.20	5.22	5.03	5.37	5.20	4.90	0.08	0.93	0.01 L	0.21
Albumin, g/dL	2.3 to 4.0	4.06	3.98	3.98	4.03	4.03	3.88	0.09	0.72	0.46	0.70
Light pig		4.02	3.83	3.97	3.90	3.97	3.85	0.17	0.82	0.94	0.71
Heavy pig		4.10	4.12	4.00	4.17	4.08	3.90	0.07	0.68	0.03 L	0.46
Globulin^4^, g/dL	3.9 to 6.0	1.30	1.36	1.10	1.22	1.28	1.41	0.13	0.64	0.85	0.24
Light pig		1.50	1.62	1.17	1.23	1.45	1.82	0.24	0.72	0.78	0.14
Heavy pig		1.10	1.10	1.03	1.20	1.12	1.00	0.05	0.54	0.06 L	0.48
A/G ratio^4^		3.28	3.18	3.71	3.43	3.57	3.33	0.23	0.78	0.72	0.24
Light pig		2.80	2.50	3.48	3.28	3.43	2.68	0.36	0.49	0.95	0.06
Heavy pig		3.75	3.87	3.93	3.57	3.70	3.98	0.21	0.56	0.37	0.82
Glucose, mg/dL	66 to 116	126.9	134.7	132.2	121.2	127.8	128.0	2.76	0.02	0.03 L	0.88
Light pig		126.8	132.5	131.8	118.3	127.0	124.5	4.36	0.06	0.25	0.94
Heavy pig		127.0	136.8	132.5	124.0	128.5	131.5	3.75	0.19	0.13	0.61
Cholesterol, mg/dL	81 to 134	85.0	82.8	74.5	86.5	84.5	80.6	2.32	0.11	0.01 L	0.54
Light pig		88.0	81.5	77.0	84.5	83.2	80.3	3.29	0.85	0.09 L	0.57
Heavy pig		82.0	84.2	72.0	88.5	85.8	80.8	3.30	0.05	0.02 L	0.55

^1^Each main effect represents the mean of 6 replicates (2 pigs/pen) for blood collected on day 21.

^2^In each pen, the heavy and light pig were selected for blood collection.

^3^SEM represent highest value.

^4^Light and heavy pig differ (*P *< 0.05).

^a,b^Means with different superscripts within rows and columns under their respective analyte differ (*P *< 0.05) when fines × boron is *P* < 0.10.

^L^Linear effect for dietary inclusion of fines (*P *< 0.05).

The last group of serum analytes measured were kidney and liver analytes (Table 9). All measures for alkaline phosphatase were 100 to 175 u/L above the reference range but among all kidney and liver analytes, there were no treatment effects (*P *> 0.05). This suggests no apparent renal or hepatic damage from the addition of these mycotoxin-containing corn fines at this concentration for a feeding period of 3 wk. However, there could have been possible morphology or immune responses to the dietary inclusions of the mycotoxins in this study. Reddy et al. (2018) concluded that high concentrations of DON and ZEA suppress the inflammatory response in kidneys at inclusion levels of 8,000 ppb DON and 800 ppm of ZEA. These levels are higher than the current study and no immune response parameters were measured herein. DON and ZEA at low levels were also seen to affect liver morphology (Skiepko et al., 2020).

**Table 9.** Liver and kidney analyte clinical chemistry for pigs (Exp. 2) fed varying levels of mycotoxin containing corn fines^1^^,^^2^

		0 ppm Boron			40 ppm Boron				*P*-values		
Item	Merck Reference range	Diet 1 (0% Fines)	Diet 2 (10% Fines)	Diet 3 (20% Fines)	Diet 4 (0% Fines)	Diet 5 (10% Fines)	Diet 6 (20% Fines)	SEM^3^	Boron	Fines	Fines*Boron
BUN^4^, mg/dL	8.2 to 25	11.2	11.3	11.5	10.8	13.0	13.0	0.77	0.14	0.20	0.36
Light pig		10.0	8.8	10.0	9.7	10.8	11.8	1.03	0.18	0.49	0.46
Heavy pig		12.3	13.8	13.0	12.0	15.2	14.2	1.13	0.44	0.14	0.72
Creatinine, mg/dL	0.8 to 2.3	0.97	1.00	0.95	0.99	0.91	0.98	0.03	0.57	0.70	0.09
Light pig		1.00	1.03	0.92	1.00	0.92	0.95	0.05	0.49	0.39	0.28
Heavy pig		0.93	0.97	0.98	0.98	0.90	1.00	0.04	1.00	0.32	0.30
BUN/Creatinine ratio^4^		11.6	11.5	12.4	11.1	14.3	13.5	0.84	0.10	0.10	0.15
Light pig		10.2	8.8	11.5	9.7	12.0	12.7	1.11	0.17	0.15	0.28
Heavy pig		13.0	14.2	13.3	12.5	16.7	14.3	1.31	0.36	0.15	0.53
Alkaline Phosphatase, U/L	41 to 176	287.7	335.5	344.3	319.8	300.2	348.2	28.90	0.99	0.33	0.51
Light pig		282.5	339.2	426.7	282.7	300	320.7	34.89	0.11	0.05 ^L^	0.33
Heavy pig		292.8	331.8	262	356.8	300.3	375.7	39.43	0.15	0.97	0.20
SGOT/AST^4^^,^^5^, U/L	15 to 55	39.3	45.3	37.3	42.6	37.6	45.7	4.98	0.75	0.99	0.27
Light pig		46.3	56.8	35.8	43.3	43.5	54.2	8.79	0.93	0.79	0.21
Heavy pig		32.2	33.8	38.8	41.8	31.7	37.2	4.79	0.62	0.52	0.39
Total bilirubin, mg/dL	0 to 0.5	0.23	0.23	0.22	0.21	0.22	0.23	0.02	0.83	0.97	0.78
Light pig		0.22	0.25	0.22	0.23	0.24	0.23	0.03	0.82	0.79	0.87
Heavy pig		0.23	0.20	0.22	0.20	0.20	0.23	0.01	0.45	0.30	0.22

^1^Each main effect represents the mean of 6 replicates (2 pigs/pen) for blood collected on day 21.

^2^In each pen, the heavy and light pig were selected for blood collection.

^3^SEM represent highest value.

^4^Light and heavy pig differ (*P *< 0.05).

^5^SGOT/AST = aspartate transaminase.

In summary, with regard to the clinical chemistry values that are reported in Tables 7–9, while there were statistically significant differences observed occasionally, those differences were relatively small on a percentage change basis. Further, when comparing the values to the Merck Index (2006) reference ranges, the changes were never such that a value for control pigs fed no mycotoxin-containing fines was within the reference range and the value for the pigs fed the highest concentration of mycotoxin-containing fines was outside the reference range. This suggests when the levels of mycotoxins fed within this study for this period of time are encountered in production situations that normal clinical chemistry analytes are not a viable biomarker for the mycotoxins. Furthermore, when examining the values in Tables 7–9 and comparing to the reference range, it can be seen that the entire set of values are often near the top or the bottom of the reference range (e.g., Ca and P) and that, at times, all values are below (e.g., globulin) or well above (e.g., alkaline phosphatase) the reference range. Thus, while published clinical chemistry reference ranges are useful tools, when it is considered that pigs used in this study were all in good health and that the mean growth rate for all dietary treatments was more than 15% above the growth rate listed for pigs in this weight range (NRC, 2012), use of a single analyte in reference ranges as a measure of well being should be used with caution.

## CONCLUSION

In conclusion, when pigs are given a choice, they have the ability to discern between diets containing these mixed mycotoxins at different inclusion levels. Yet, pigs do not have different growth performance using the particular mycotoxins and concentrations within the framework of this assessment.
